# Topical Vitamin C Promotes the Recovery of Corneal Alkali Burns in Mice

**DOI:** 10.1155/2021/2406646

**Published:** 2021-12-23

**Authors:** Min Li, Zufeng Chen, Lin Liu, Xiaoyun Ma, Jun Zou

**Affiliations:** ^1^Department of Ophthalmology, Shanghai Tenth People's Hospital, Tongji University, Shanghai, China; ^2^Department of Ophthalmology, Shanghai University of Medicine & Health Sciences Affiliated Zhoupu Hospital, Shanghai, China

## Abstract

**Background:**

Vitamin C (Vc) has been found to promote corneal wound healing after alkali burns. However, the specific mechanism and functional modes are still unclear. The present study sought to assess the mechanisms of Vc function on corneal alkali burns.

**Methods:**

Eighty BALB/c mice were divided into four groups: a normal group without alkali injury (*n* = 10), an alkali injury group without any treatment (1-day group, *n* = 10), a Vc group treated with topical 10% Vc (Vc group, *n* = 30), and a control group treated with topical sterile water (control group, *n* = 30). Except in the blank control group, the alkali injuries were induced in one eye of each mouse. The mice in the treatment group were given Vc by topical application (q 1 h for 6 days), while those in the control group were given topical sterile water. The clinical evaluations, including corneal fluorescent staining, corneal opacity, and neovascularization, were assessed on days 1, 4, 7, and 10 using slit-lamp microscopy. Ten mice at each time point were sacrificed. The protein expressions in the corneas of p63, PCNA, CK3, MPO, CD31, and *α*-SMA were detected by immunohistochemistry to examine the corneal epithelial stem cells, corneal epithelium wound healing, corneal stroma inflammation, neovascularization, and fibrosis.

**Results:**

The scores of the corneal epithelium defects, corneal neovascularization, and corneal opacities in the Vc group were significantly decreased compared to the control group on day 10. We found that Vc promoted the activation of the corneal epithelial stem cells as shown by a higher number of p63-positive and PCNA-positive cells and an increased CK3 expression when compared with the control group (*p* < 0.001). The central corneal re-epithelialization was completed by day 10. Moreover, Vc inhibited MPO, CD31, and *α*-SMA expressions. These results first indicated that the frequent use of topical Vc in the first 6 days of corneal alkali burns alleviated corneal inflammatory cell infiltration, activated corneal epithelial stem cell activity, and reduced corneal neovascularization and fibrosis within 10 days.

**Conclusions:**

The study, therefore, showed the therapeutic benefits of Vc on corneal alkali burns and provided new insight into the mechanisms of Vc regulation on corneal wound healing.

## 1. Introduction

Acute ocular chemical burns are ophthalmic emergencies accounting for 11.5% to 22.1% of ocular injuries and are among the most difficult eye injuries to treat and manage [1, 2]. Alkali burns account for nearly 60% of all chemical ocular injuries. At the moment of contact with the ocular surface, the alkali can immediately damage the cornea and conjunctiva. Alkali destroys the proteoglycan ground substance and the highly ordered collagen bundles in the corneal stroma, with the continuous production of proteases from the damaged tissue, causing extensive tissue damage [3]. Severe burns can lead to slow epithelial regeneration, corneal ulceration, corneal neovascularization, and corneal opacification. They may cause permanent visual impairment or even corneal blindness. Many medical and surgical treatments are applied to treat alkali burns. The surgical treatment of ocular burns includes amniotic membrane transplantation, limbal stem cell transplantation, and penetrating keratoplasty. Medications such as citrate [4], tetracycline [5], corticosteroids [6], and medroxyprogesterone [7] have been shown to benefit corneal wound healing.

Vitamin C (Vc) has been used since 1951 for the treatment of cornea and conjunctiva burns caused by alkaline agents [8]. Several previous studies have reported that topical Vc application increases the concentration of Vc in the aqueous humor, reduces the incidence of corneal perforation, and inhibits corneal neovascularization [9–13]. However, these studies only observed the therapeutic effect of Vc. Lee found that Vc reduced the concentrations of matrix metalloproteinase-9 and vascular endothelial growth factor in the corneas of a silk suture model of corneal neovascularization [14]. Vc is also well-known for its effect on promoting cell proliferation [15]. Furthermore, Vc has been shown to enhance the stemness of mouse corneal epithelial progenitor cells (TKE2) by promoting extracellular matrix production [16]. However, the therapeutic mechanism of vitamins on the corneal alkali burn is still unclear. Our study, therefore, determined the frequency of topical Vc application and characterized the molecular mechanism of Vc in the treatment of corneal epithelium defects, corneal opacifications, inflammation, neovascularization, and corneal stromal fibrosis in a mouse model.

## 2. Methods

### 2.1. The Animal Model of Corneal Alkali Injury

A total of 80 eight-week-old male BALB/c mice (Animal Center of Shanghai Laboratory, Shanghai, China) received a minimum of 1 week of acclimation. The number of mice per group was calculated using a power equation method introduced in the former study [17]. There were 10 mice in each time point per group, while the minimum number of mice was 8.4 according to the calculation results. The mice were divided into four groups: a normal group without alkali injury (*n* = 10), an alkali injury group without any treatment (1-day group, *n* = 10), a Vc group treated with topical 10% Vc (Vc group, *n* = 30), and a control group treated with topical sterile water (control group, *n* = 30). The corneal alkali injury model was established in 70 mice of the three groups, except the normal group. To establish the corneal alkali injury model, pentobarbital (40 mg/kg) was injected intraperitoneally in the mice. Topical anesthesia (0.5% proparacaine hydrochloride; Alcon, Fort Worth, TX, USA) was also used before corneal alkali injury. The alkaline burn was induced onto one cornea alone of each mouse. The corneal alkali burn models were produced with a 2 mm diameter round filter paper soaked with 1 *μ*L of 1 M NaOH and placed on the surface of the central cornea for 30 s. After the filter paper was removed from the cornea, the cornea was flushed with sterilized physiological saline water for 1 min. This protocol was performed on the right eye of the mice.

### 2.2. Animal Treatments

After one day of alkali injury, the mice in the Vc and control groups received topical Vc treatment or topical sterile water treatment, respectively. For the Vc treatment group, the mice were given Vc by the topical application of eye drops prepared with sterile water once an hour (10%, eight times per day). The control group was treated with topical sterile water. Treatments were conducted 24 h after the corneal alkali injury and were administered daily for 7 days. To prevent heterogeneity between the groups and achieve more reliable results, the mice that had an excessive or insufficient corneal burn were excluded from the experiments on the following second day. The exclusion criteria were a poor extent of alkali burns and corneal perforation [12].

### 2.3. Biological Microscopic Observation

The photography of the corneas using slit-lamp microscopy (Carl Zeiss, Jena, Germany) was performed on days 1, 4, 7, and 10 after the corneal alkali injury. Grading was performed by two experienced ophthalmologists who were blinded to the assignment of the mice.

The degree of corneal epithelial defects was detected by a fluorescein test. Briefly, 5 *μ*L of 0.1% sodium fluorescein solution (Tianjin Jingming New Technological Development, Tianjin, China) was instilled in each eye of the mice. Cobalt blue light was used to examine the corneal epithelial defect. The images were then captured using a digital camera. The epithelial defects were scored as follows: 0 (a cornea with no staining), 0.5 (a cornea with slight punctate staining), 1 (a cornea with diffuse punctate staining), 2 (a cornea with diffuse staining, area <1/3), 3 (a cornea with diffuse staining, area >1/3), and 4 (a cornea staining area >2/3) [13].

The corneal opacity was scored as follows: 0 (completely clear), 1 (slight opacity, the details of the iris were visible), 2 (moderate opacity, the iris vessels were still visible), 3 (opaque, the pupil was visible with haze), and 4 (opaque, the pupil was not visible) [13, 18].

Corneal neovascularization (CNV) was graded as follows: 0 (no neovessel), 1 (neovessels in the peripheral cornea, <1/3 area of the corneal), 2 (neovessels, <2/3 area of the cornea), and 3 (neovessels, the entire cornea) [19].

### 2.4. Histology and Immunohistochemistry

Mice were sacrificed at 1, 4, 7, and 10 days after injury. The eyeballs were removed, fixed in 10% buffered formalin, and embedded in paraffin. Continuous sections were made from the corneal limbus to the center, parallel to the optic papilla sagittal position [20]. Samples were cut into 4 *μ*m slices and stained with hematoxylin and eosin (HE). Three representative sections per eye were analyzed with a microscope. Each cross-section was examined with ×400 magnification. For immunohistochemical analyses, the samples were blocked in 3% goat serum (AR1010; Boster, Pleasanton, CA, USA) for 30 min at room temperature, and subsequently, they were incubated with primary antibodies diluted in phosphate-buffered saline (PBS) overnight at 4°C. The samples were then incubated with a biotinylated secondary IgG antibody (1 : 1, K5007; Dako, Carpenteria, CA, USA) for 50 min at room temperature. The following primary antibodies were used for the immunohistochemical analyses: p63 (1 : 1,200, AB124762; Abcam, Cambridge, UK), CK3 (1 : 100, ab77869; Abcam), proliferating cell nuclear antigen (PCNA) (1 : 2,000, GB13010-1; Servicebio, Wuhan, China), alpha-smooth muscle actin (*α*-SMA) (1 : 500, GB13044; Servicebio), CD31 (1 : 100; Sangon Biotech, Shanghai, China), and myeloperoxidase (MPO) (1 : 500, GB11224; Servicebio). The mean density (IOD/area) (IOD, integral optical density) was analyzed using Image-Pro Plus 6.0 (Media Cybernetics, Bethesda, MD, USA).

### 2.5. Statistical Analysis

Data were expressed as the mean ± SEM (standard error of the mean). All analyses were performed by Prism 5 (GraphPad Software, LaJolla, CA, USA). Statistical analyses were performed using the Student's nonpaired *t*-test. A value of *p* < 0.05 was deemed significant.

## 3. Results

### 3.1. Vc Ameliorated the Clinical Signs of Alkali-Burned Corneas

**Figure 1:**
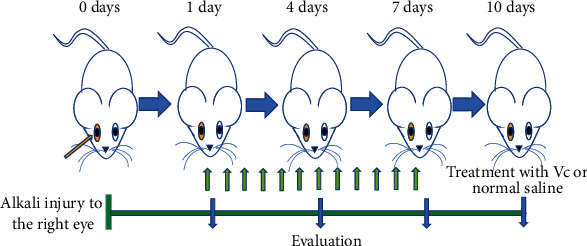
Schematic representation of the time course for the experiments. Days 0 to 10; time after alkali injury (day). Day 0; corneal alkali burns were created in the right eye of mice. From day 1 to 7, the mice were treated with vitamin C. Biomicroscopic and histopathological examinations were conducted on days 1, 4, 7, and 10 after the alkali burns.

**Figure 2:**
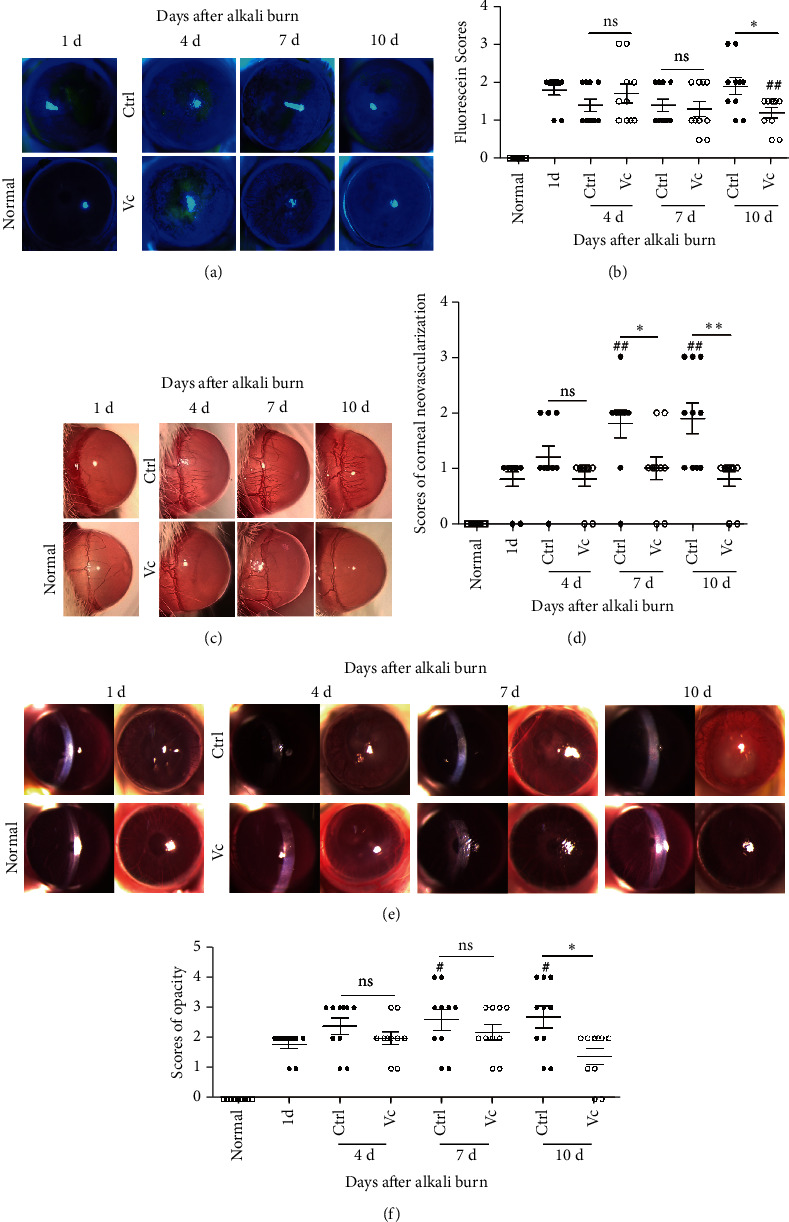
Clinical analysis of the alkali-burned corneas treated with vitamin C (Vc). (a) The corneas of the alkali-burned mice were stained with sodium fluorescein. (b) The results of the epithelial damage scores expressed as the mean ± SEM. (c) Representative images of neovascularization in the two groups. (d) The results of corneal neovascularization scores expressed as the mean ± SEM. (e) Representative images of corneal opacity in different groups. (f) The results of corneal opacity scores, expressed as the mean ± SEM. Abbreviations: Ctrl, control group (sterile water group); Vc, Vitamin C group; ns, no significant difference between the indicated groups. ^*∗*^Means the comparison of Vc group and control group. ^#^ Means the comparison on day 1. ^*∗*^*p* < 0.05, ^*∗∗*^*p* < 0.01, and ^##^*p* < 0.01. Scale bar = 50 *μ*m.

The corneal alkali burn models were successfully produced in 80 mice. One day after the alkali injury, the corneas were treated daily with Vc or sterile water (Figure 1). The clinical signs on days 1, 4, 7, and 10 after the alkali injury are shown in Figure 2. From days 1 to 7, the epithelial defect staining was diffuse, and the injury area was smaller than 1/3 of the total cornea in the control group (Figure 2(a)). In the Vc group, the epithelial defects of one mouse involved slight punctate staining on day 7 (Figure 2(a)). On day 10, the staining was between slight-to-diffuse punctate, and the scores were improved compared to day 1 (*p* < 0.01) in the Vc group. The defect score in the Vc group was significantly reduced compared to the control group (*p* < 0.05) (Figure 2(b)).

CNV progressively increased after the injury and reached a peak on days 7 and 10, covering between 1/3 and 2/3 of the total cornea (Figure 2(c)). In the Vc group, the neovessels covered less than 1/3 of the cornea at day 7, and half of the mice had no obvious neovessels on day 10 (Figure 2(c)). The scores on days 7 and 10 were significantly lower than those of the control group (*p* < 0.05) (Figure 2(d)).

Moderate corneal opacity occurred on day 1 and progressed afterwards with a faint pupil seen on day 10 (Figure 2(e)). With Vc treatment, the corneal opacification and scores improved, with the iris well-visualized on day 10 (Figures 2(e) and 2(f)).

### 3.2. Vc Promotes the Stemness and Differentiation Ability of Corneal Epithelial Stem Cells

The corneal epithelial stem cells differentiate into the corneal epithelial cells (CECs), which is essential for maintaining a clear and transparent cornea. Under pathological conditions, the CECs change into epithelioid cells and show a decreased expression of the cornea-specific marker, CK3, leading to the loss of corneal transparency [21]. As important markers of the corneal epithelial stem cells, the expressions of p63 and PCNA were evaluated by immunohistochemical analyses.

**Figure 3:**
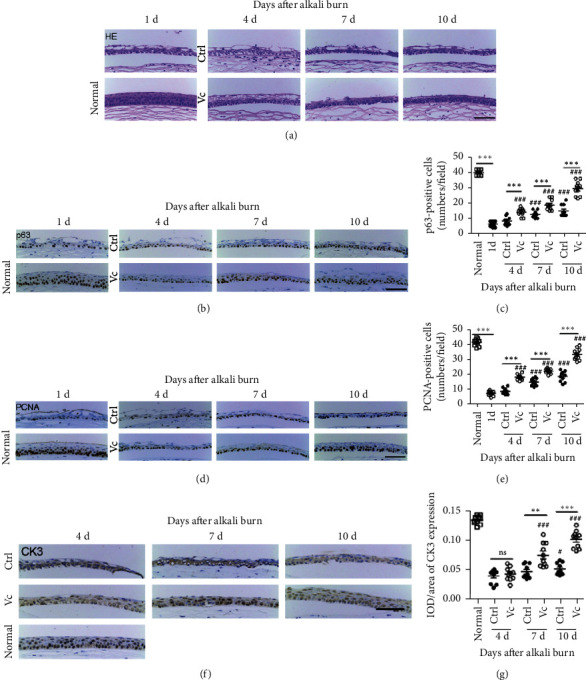
Vitamin C promotes the stemness of corneal epithelial stem cells and increases the CK3 expression *in vivo*. (a) Representative images of hematoxylin and eosin staining of injured corneas. (b) Immunohistochemical analyses of p63 expression. (c) The number of p63-positive cells in both groups. (d) Immunohistochemical analyses of proliferating cell nuclear antigen (PCNA) expression. (e) The number of PCNA-positive cells in both groups. (f) Immunohistochemical analyses of CK3 expression. (g) The IOD/area of CK3 in the cornea. IOD refers to the integral optical density. Abbreviations: Ctrl, control group (sterile water group); Vc, vitamin C group; ns, no significant difference between the indicated groups. ^*∗*^ means the comparison of Vc group and control group. ## and ### means the comparison with day 1. ## in G means the comparison with the Vc group on day 4. ^*∗*^*p* < 0.05, ^*∗∗*^*p* < 0.01, ^*∗∗∗*^*p* < 0.001, ^##^*p* < 0.01, and ^###^*p* < 0.001. Scale bar = 50 *μ*m.

The HE-stained sections of the normal corneas showed distinct epithelial layers and organized corneas (epithelium and stroma). Corneal epithelial denudation and vacuolation were observed at the center of the cornea after the alkali burns (Figure 3(a)). In the control group, the epithelium was disorganized, and the regeneration of the corneal epithelium was not completed by day 10. In contrast, the corneal epithelial structure was gradually repaired, the epithelial layers were gradually increased, and the central corneal re-epithelialization was completed by day 10 in the Vc group. The alkali burns destroyed the mice's corneal epithelium. The number of p63-positive cells was significantly decreased on day 1 when compared with the normal cornea (6.20 ± 0.51vs. 40.00 ± 0.52, *p* < 0.001, *n* = 10). The positive number was gradually restored and was higher in the Vc group when compared to the control group after 4 days (14.10 ± 0.83 vs. 8.300 ± 0.87, *p* < 0.001, *n* = 10), 7 days (18.80 ± 1.10 vs. 12.70 ± 0.68, *p* < 0.001, *n* = 10), and 10 days (29.40 ± 1.52 vs. 15.00 ± 1.16, *p* < 0.001, *n* = 10) (Figures 3(b) and 3(c)). The pattern of PCNA expression was the same as p63 (Figures 3(d) and 3(e)).

As it takes time for the corneal epithelial stem cells to differentiate into CECs, we detected the expression of CK3 on day 4. The expression of CK3 was not improved greatly by day 7 when compared with day 4 in the control group (Figure 3(f)). On days 7 and 10, the IOD/area of CK3 expression was apparently higher in the Vc group (*p* < 0.01) (Figure 3(g)). The presence of CK3 increased in the epithelium of the Vc group on days 7 and 10 when compared with that on day 4 (*p* < 0.001).

### 3.3. Vc Accelerates Corneal Epithelial Wound Healing by Attenuating the Corneal Inflammation Response

**Figure 4:**
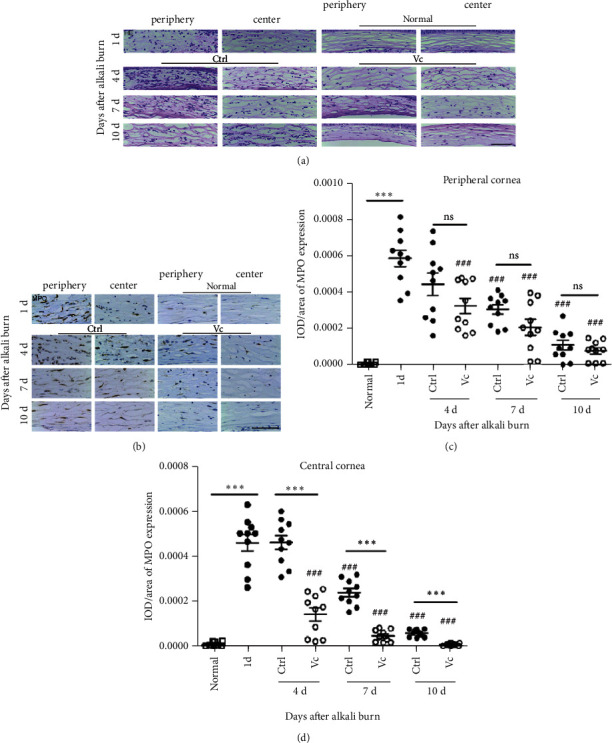
Vitamin C inhibits the infiltration of the inflammatory cells. (a) The hematoxylin and eosin stains of the corneas of the two groups. (b) MPO immunohistochemical staining of the center and periphery of the cornea. (c, d) IOD/area of the MPO in the central and peripheral areas of the cornea. Abbreviations: Ctrl, control group (sterile water group); Vc, Vitamin C group; ns, no significant difference between the indicated groups. ^*∗*^Means the comparison of Vc group and control group. ^#^Means the comparison with day 1. ^*∗*^*p* < 0.05, ^*∗∗*^*p* < 0.01, and ^#^*p* < 0.05. Scale bar = 50 *μ*m. IOD means integral optical density, and MPO is myeloperoxidase.

To investigate the anti-inflammatory effects of Vc, we detected the expression of MPO with immunohistochemical and hematoxylin & eosin (HE) staining. The normal corneal stroma was well-aligned, where there were almost no inflammatory cells (Figure 4(a)). The inflammatory cell infiltration was observed in the corneas after the alkali injury. On day 1, the peripheral corneal stroma of the alkali-burned cornea had many inflammatory cells staining in both groups (Figures 4(a) and 4(b)).

Neutrophil infiltration was assessed by the level of MPO protein. MPO in the central corneal stroma peaked on days 1 and 4 and reduced to 51.11% of the peak level by day 7. The MPO was lower in the Vc group compared with that of the control group after 4 days, (1.40 × 10^−4^ ± 0.29 × 10^−4^ vs. 4.59 × 10^−4^ ± 0.31 × 10^−4^, *p* < 0.001, *n* = 10), 7 days (0.44 × 10^−4^ ± 0.01 × 10^−4^ vs. 2.37 × 10^−4^ ± 0.18 × 10^−4^, *p* < 0.001, *n* = 10), and 10 days (0.06 × 10^−4^ ± 0.02 × 10^−4^ vs. 0.56 × 10^−4^ ± 0.05 × 10^−4^, *p* < 0.001, *n* = 10) (Figures 4(a)–4(c)).

Very similar results of MPO-positive neutrophils in the periphery of the cornea were observed in the two groups (Figure 4(c)). However, the expression of MPO in the central corneal stroma in the Vc group was much less than that in the sterile water group from day 4 to 10 (*p* < 0.05) (Figure 4(d)).

### 3.4. Vc Inhibits the Expression of *α*-SMA and CD31 in Alkali Burned Corneas in Mice

**Figure 5:**
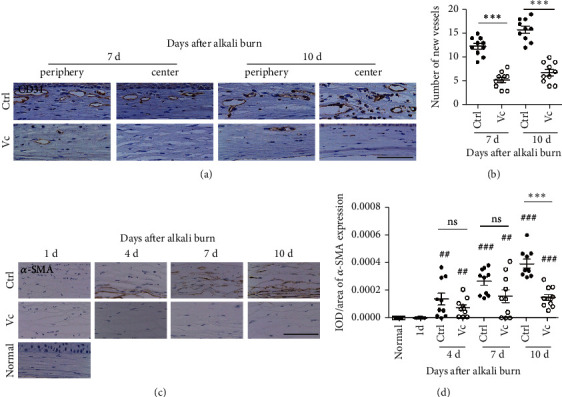
Quantification of neovascularization and the opacity of alkali-burned mouse corneas. (a) Representative CD31 staining images in the alkali-burned corneas on days 7 and 10 after injury. (b) The number of CD31-positive new vessels in the two groups. (c) The expression of the smooth muscle actin (SMA) of the two groups from day 1 to day 10 after immunohistochemical staining. (d) The IOD/areas of *α*-SMA in the corneal stromas of the two groups. IOD means integral optical density. Abbreviations: Ctrl, control group (sterile water group); Vc, vitamin C group; ns, no significant difference between the indicated groups. ^*∗*^Means the comparison of Vc group and control group. ^#^ Means the comparison with day 1. ^*∗∗*^*p* < 0.01, ^#^*p* < 0.05, and ^##^*p* < 0.01. Scale bar = 50 *μ*m.

From day 7 to 10 after the corneal injury, a greater number of neovascular structures were observed in the cornea in the control group, and the expression of CD31 in the Vc group was lower than that of the control group (Figure 5(a)). The numbers of the new vessels in the corneas in the Vc group were less than those in the control group on days 7 and 10 (*p* < 0.001) (Figure 5(b)).

The *α*-SMA is a key factor for corneal scar formation, which is rarely observed in the normal corneas. There was the expression of *α*-SMA in the control group on day 10 after alkali burn (Figure 5(c)). The Vc group, however, showed little expression of *α*-SMA on day 10 after the alkali burn, and the IOD/area of *α*-SMA in the Vc group was lower than that of the control group (*p* < 0.001) (Figure 5(d)).

## 4. Discussion

The integrity of the corneal epithelium plays an important role in maintaining the transparency of the corneas and the visual function of the eyes. Corneal alkali burns cause damage to the ocular surfaces and the limbal stem cells (LSCs), leading to corneal epithelial loss, corneal neovascularization, and corneal opacity [4, 19, 22]. VC has been proved to play a protective role in the repair of corneal disease repair by UV irradiation [23], corneal neovascularization [14], and inflammation [23]. In the present study, Vc was administered mainly by the topical eye drops and intraperitoneal injection, however, the administration time and the observation time were different. Our results showed that the frequent topical application of Vc to the alkali-burned corneas (q 1 h for 6 days) quickly inhibited the infiltration of the inflammatory cells, promoted the recovery of LSCs, completed the repair of the corneal epithelium, and reduced the formation of corneal neovascularization and the degree of corneal opacity within 10 days.

Traditionally, corneal LSCs are considered slow cycling but have the potential for self-renewal, proliferation, and differentiation in response to appropriate stimulation. The corneal stem cells can produce transient amplifying cells (TACs) that have high proliferation and differentiation ability. TACs migrate apically toward the center of the cornea and replace the lost CECs [24–26]. The corneal alkali burns damage the normal function of the LSCs and cause limbal stem cell dysfunction (LSCD). LSCD causes a variety of ocular surface dysfunctions that are unable to maintain physiological corneal epithelial repair, leading to continuous corneal epithelial loss, corneal neovascularization, chronic inflammation, corneal opacity, and visual loss [27]. In this study, we observed that the corneal surface function was disordered, and the corneal epithelium healed for an extended period of time, accompanied by a large number of corneal neovascularizations and corneal opacities in the control group. After 1 week of Vc treatment, the ocular surface condition of mice improved, the healing time of corneal epithelium was shorter, the corneal neovascularization decreased, and the degree of corneal opacity reduced. The results showed that Vc treatment improved corneal epithelial healing and promoted the recovery of ocular surface function of LSCD mice after alkali burns.

The p63 molecule has been widely recognized as a marker of LSCs. It is a member of the p53 family and plays a key role in cell cycle regulation [24, 28]. PCNA is a kind of polypeptide synthesized or expressed in the proliferating cell nuclei. Its expression and synthesis are related to the cell cycle. PCNA marks the LSCs and TACs in the corneal epithelium [29]. CK3 is a cytoskeleton component composed of intermediate filament proteins encoded by the highly conserved CK3 gene that plays a vital role in the structural stability of CECs. Previous studies have shown that ascorbic acid can promote the stemness of the corneal epithelial stem cells. Investigators scraped the corneal epithelium in the limbal region, and ascorbic acid was used in the eye drops. The results showed that ascorbic acid accelerated the corneal epithelial wound healing and increased p63 expression *in vivo* at 48 h after epithelial debridement [16]. In our study, we established a corneal alkali burn model. Similarly, we also observed a significant increase of p63 and PCNA in the Vc-treated corneas from day 4 to day 10 after burning. HE staining showed that the regeneration of corneal epithelium in the Vc-treated group completed on day 10, with a significantly increased expression of CK3. It may explain the acceleration of the corneal epithelial wound healing caused by Vc *in vivo* and may promote the proliferation and differentiation of the corneal epithelial stem cells into the corneal epithelial cells.

In the early stage of alkali burns, a large number of neutrophils infiltrate and release collagenase, thus causing severe damage to the cornea. The tissue inflammatory microenvironment can affect the function of the stem cells [30]. In the cornea, the inflammatory microenvironment may affect the differentiation and regeneration of the limbal stem cells. Persistent inflammation on the ocular surface may affect the survival of the limbal stem cell transplantation [31–33]. Vc shows antioxidant protection during the inflammatory response to scavenge the oxygen free radicals and metabolites, such as myeloperoxidase produced by the inflammatory cells [34]. Previous studies have suggested that Vc plays a protective role during the inflammatory episodes in the eye, where it inhibits damage to the epithelium and matrix around the wounds [35, 36]. In addition, Vc favorably modulates the inflammatory cell functions [37]. Kasetsuwan et al. [34] established the models of photorefractive keratectomy using an excimer laser on New Zealand white rabbits and treated them with topical 10% Vc. The results showed that the Vc treatment significantly reduced the number of polymorphonuclear cells and lipid peroxidation after 24 h when compared with the control group. Our results showed that from day 4, there was less infiltration of the inflammatory cells in the alkali-burned areas of the Vc-treated cornea, and the IOD/area of MPO expression in the central cornea was significantly lower than that in the sterile water-treated group. It showed that Vc inhibited the inflammatory response after alkali burns, which contributed to the recovery of the corneal stem cell function because the proliferation of the corneal stem cells was observed on day 4.

**Figure 6:**
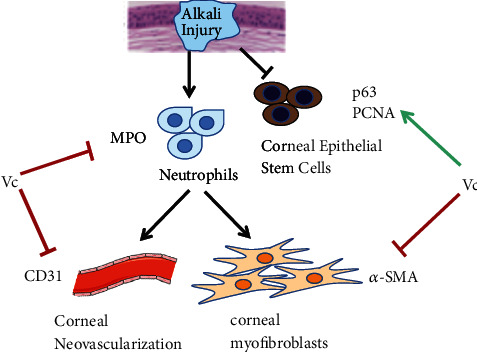
The mechanism of Vc for promoting corneal epithelial wound healing and inhibiting corneal neovascularization, corneal inflammation, and corneal fibrosis. “⟶” means promoting effect. “·” means inhibiting effect.

Previous studies have reported that inflammation is an important factor in the development of CNV [38], and CNV can aggravate corneal scar formation and reduce vision [39]. CD31 marks the vascular endothelial cells and reflects the level of angiogenesis [40]. The alkali burns not only cause the corneal epithelial damage but also cause the corneal stroma damage. Once the corneal stroma is injured, the keratocytes near the wound are activated and transformed into myofibroblasts, which results in corneal scar formation and affects corneal transparency [41]. The *α*-SMA reflects the activation of corneal myofibroblasts and the severity of corneal scars [42]. In the present study, we showed that Vc inhibited the infiltration of the inflammatory cells in the alkali-burned areas of the cornea on day 4. At the same time, it was observed that the Vc treatment significantly inhibited the growth of corneal neovascularization, and the number of CD31-positive vessels in the corneas of the mice treated with Vc was significantly lower than that in the sterile water group. On day 10, the expressions of *α*-SMA in the corneas of the Vc group were significantly lower, and at the same time, the corneal turbidity was significantly reduced. We, therefore, suggest that Vc inhibited the production of corneal neovascularization by inhibiting inflammation, and the decrease of angiogenesis was beneficial to the reduction of corneal fibrosis. The mechanism of Vc with corneal alkali burns is summarized in Figure 6.

## 5. Conclusion

Our study showed that the frequent topical treatment of Vc with corneal alkali burns in the early stage promoted the proliferation and differentiation of the corneal stem cells by maintaining the homeostasis of the corneal stromal microenvironment, alleviated corneal inflammatory, and reduced the corneal neovascularizition, thereby promoting the healing of the corneal alkali burns in mice. In the future, this study also needs an in-depth verification of the mechanism of action, which is now in progress.

## Data Availability

The data used to support the results of this study are included within the article.
